# Supplementary material for: The impact of introduction of the 10-valent pneumococcal conjugate vaccine on pneumococcal carriage in Nigeria
Source: Nat Commun. 2023 May 9;14:2666. doi: 10.1038/s41467-023-38277-z (PMC10169786; doi:10.1038/s41467-023-38277-z)
Supplement: Supplementary file 1 — Supplementary Information [file 41467_2023_38277_MOESM1_ESM.pdf]

Supplement

The impact of introduction of the 10-valent pneumococcal conjugate vaccine on pneumococcal carriage in Nigeria

Authors

Aishatu L Adamu[1,2,3*], J. Ojal[1,2], Isa A. Abubakar[3], Kofo Odeyemi[4], Musa M. Bello[3], Christy A.N. Okoromah[5], Boniface Karia[1], Angela Karani[1], Donald. Akech[1], Victor Inem[4], J. Anthony G Scott[1,2], Ifedayo M.O. Adetifa[1,2,5,6]

[1]KEMRI-Wellcome Trust Research Programme, Kilifi, Kenya

[2]Department of Infectious Diseases Epidemiology, London School of Hygiene & Tropical Medicine, UK

[3]Department of Community Medicine, College of Health Sciences, Bayero University, Kano/Aminu Kano Teaching Hospital, Kano, Nigeria

[4]Department of Community Medicine and Primary Care, College of Medicine, University of Lagos, Lagos, Nigeria

[5]Department of Paediatrics and Child Health, College of Medicine, University of Lagos, Lagos, Nigeria.

[6]Nigeria Centre for Disease Control, Abuja, Nigeria

*Corresponding author

These authors jointly supervised this work: JAGS and IMOA

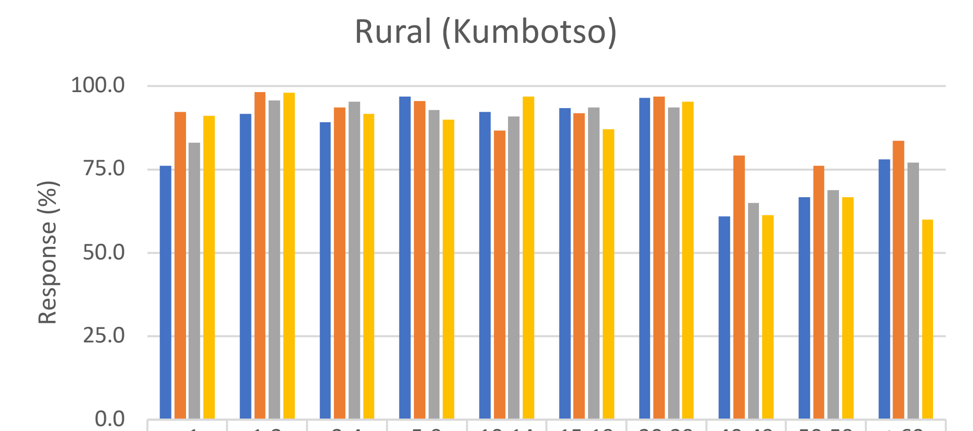

	<1	1-2	3-4	5-9	10-14	15-19	20-39	40-49	50-59	≥60
2017	76.0	91.7	89.1	96.9	92.3	93.3	96.4	61.0	66.7	78.0
2018	92.3	98.2	93.6	95.5	86.7	91.8	96.9	79.1	76.0	83.6
2019	83.0	95.6	95.3	92.9	90.8	93.6	93.6	65.0	68.8	77.0
2020	91.0	98.0	91.7	90.0	96.9	87.0	95.4	61.3	66.7	60.0

2017 2018 2019 2020

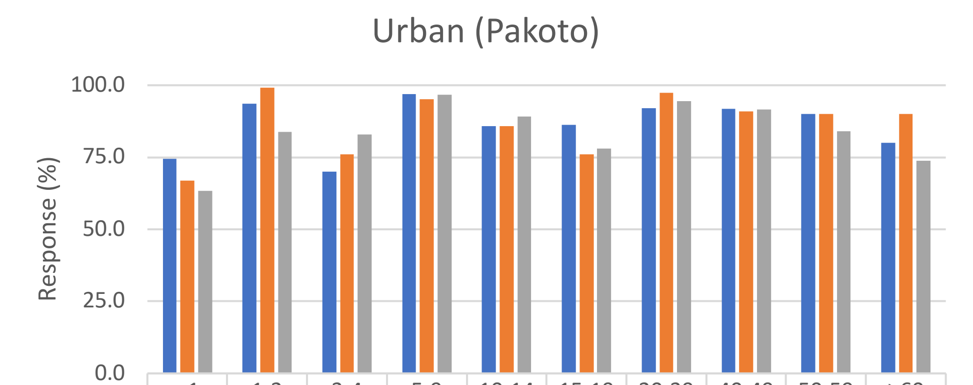

	<1	1-2	3-4	5-9	10-14	15-19	20-39	40-49	50-59	≥60
2018	74.5	93.6	70.0	96.9	85.7	86.4	92.0	91.8	90.0	80.0
2019	67.0	99.1	76.0	95.2	85.8	76.0	97.3	90.9	90.0	90.0
2020	63.3	83.8	83.0	96.7	89.2	78.0	94.5	91.7	84.0	73.8

2018 2019 2020

Supplementary Fig. 1: **Annual participation in carriage surveys.** Proportion of invited participants in sampled age groups that consented to be swabbed by survey year in the rural and urban sites.

Supplement Table 1: Numbers of participants invited*, consented, and swabbed in each carriage survey in the rural site

Age group	swabbed	invited	consented	swabbed	invited	consented	swabbed	invited	consented	swabbed	invited	consented	swabbed
	2016**	2017			2018			2019			2020		
**Kumbotso (rural)**													
<1	73	100	76	71	130	120	108	100	83	82	100	91	89
1-2	132	120	110	105	110	108	101	160	153	147	150	147	141
3-4	91	110	98	88	110	103	95	150	143	133	120	110	106
5-9	191	130	126	116	110	105	102	140	130	124	140	126	120
10-14	81	130	120	110	150	130	119	120	109	106	160	155	149
15-19	38	120	112	107	110	101	97	110	103	96	100	87	85
20-39	154	110	106	104	130	126	125	110	103	101	130	124	122
40-49	46	100	61	61	110	87	87	80	52	52	80	49	47
50-59	30	60	40	39	100	76	75	80	55	55	60	40	40
≥60	36	100	78	78	110	92	90	100	77	76	100	60	58
**Total**	**872**	**1,080**	**927**	**879**	**1,170**	**1048**	**999**	**1,150**	**1,008**	**972**	**1,140**	**989**	**957**
**Pakoto (urban)**													
	2017**	2018			2019			2020					
<1	109	110	82	76	100	67	65	60	38	37			
1-2	141	110	103	100	110	109	105	80	67	67			
3-4	85	100	70	68	100	76	73	100	83	81			
5-9	161	130	126	119	125	119	117	120	116	116			
10-14	75	140	120	117	120	103	102	130	116	115			
15-19	20	110	95	92	100	76	76	100	78	78			
20-39	130	100	92	91	110	107	106	110	104	104			
40-49	57	110	101	100	110	100	99	120	110	110			
50-59	66	110	99	98	100	90	90	100	84	84			
≥60	75	100	80	80	110	99	99	80	59	59			
**Total**	**919**	**1120**	**968**	**941**	**1085**	**946**	**932**	**1000**	**855**	**851**			

* Age groups (school-aged children and young adolescents) with poor participation due to 'apprehension' about the swab procedure were oversampled to improve response. Participants or their caregivers occasionally made errors in reporting ages at the time of invitation. Such participants had to be reclassified to their correct age groups at the time of interview and the wrongfully assigned age group had to be resampled. ** Sampling technique was not age-stratified in this year. This was a convenient sample volunteers and results of this survey have been published. (1)

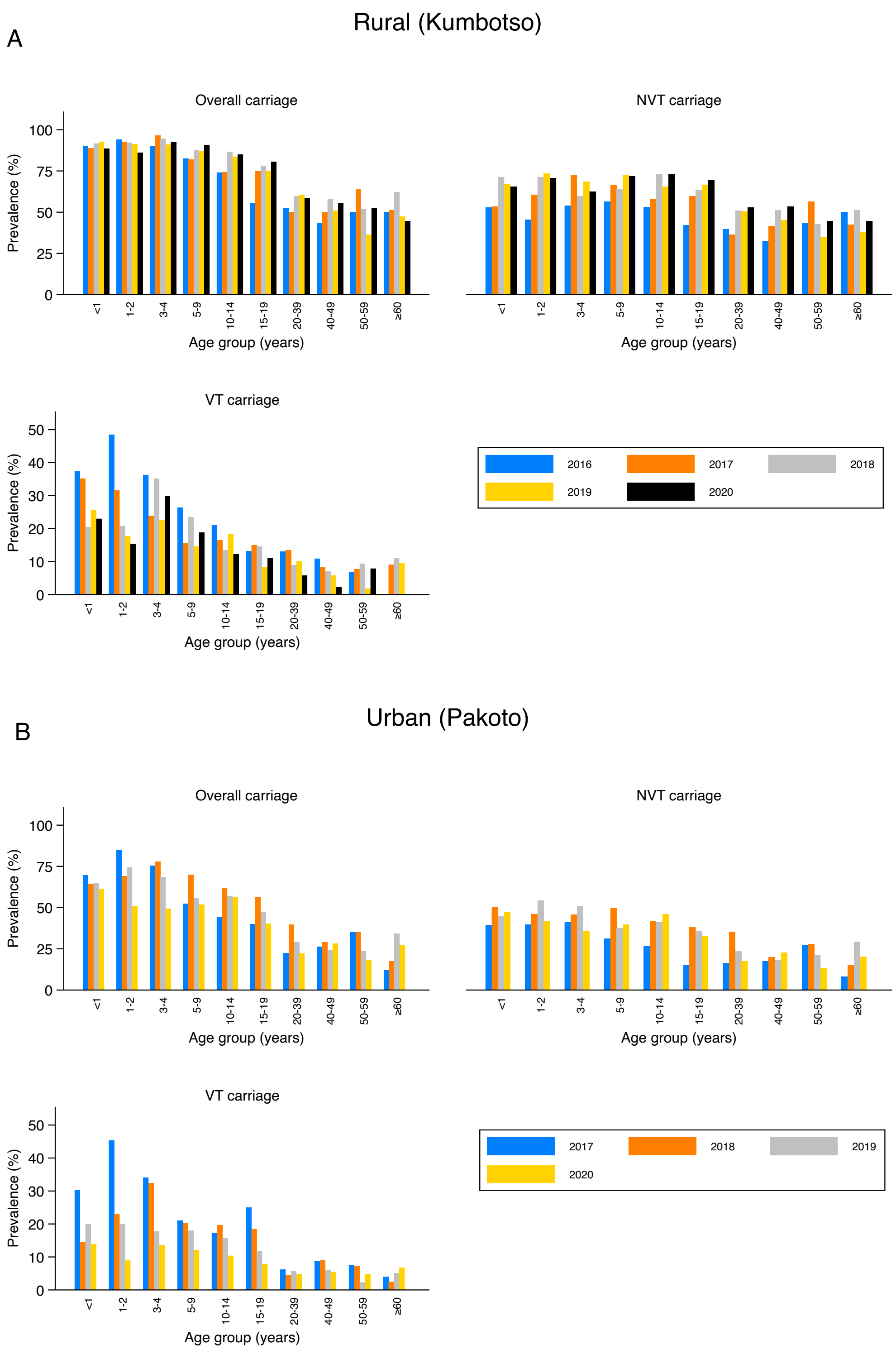

Supplementary Fig. 2: **Age-stratified annual pneumococcal carriage prevalence.** Carriage prevalence of overall, NVT and VT across sampled age groups by survey year in the rural site Note scales for VT carriage are from 0-50%.

Supplement Table 2: Annual crude serotype-specific carriage prevalence and crude odds ratio (OR) and 95% CI from logistic regression comparing carriage in the final survey (Year 5) to the baseline survey (year 1) of VTs and the commonest NVTs among children aged <5 years in Kumbotso, the rural site

Children aged <5 years in Kumbotso (rural)							
Serotype	Prevalence (95% CI)						
	Year 1	Year 2	Year 3	Year 4	Year 5	Crude OR (95% CI)	P value*
19F	16.2 (12.0,21.5)	9.9 (6.4,14.4)	6.6 (4.0,10.2)	4.9 (2.9,7.8)	5.4 (3.2,8.5)	**0.3 (0.2,0.54)**	**<0.0001**
23F	10.5 (7.1,14.9)	9.9 (6.4,14.4)	4.9 (2.8,8.1)	6.9 (4.4,10.1)	3.9 (2.1,6.7)	**0.3 (0.2,0.7)**	**0.003**
6B	5.1 (2.8,8.4)	3.4 (1.6,6.5)	3.3 (1.6,6.1)	3.8 (2.1,6.4)	3.3 (1.7,5.9)	0.7 (0.3,1.5)	0.33
14	3.0 (1.4,5.8)	1.9 (0.6,4.4)	2.3 (0.9,4.7)	1.1 (0.3,2.8)	4.5 (2.5,7.4)	1.6 (0.7,3.7)	0.29
4	2.7 (1.2,5.3)	1.1 (0.2,3.3)	0.7 (0.1,2.4)	0.8 (0.2,2.4)	0.6 (0.1,2.2)	0.2 (0.5,1.1)	0.06
9V	2.4 (1.0,4.9)	2.7 (1.1,5.5)	2.6 (1.1,5.2)	0.0 (0.0,0.0)	0.9 (0.2,2.6)	0.4 (0.1,1.5)	0.18
18C	1.7 (0.6,3.9)	1.1 (0.2,3.3)	2.0 (0.7,4.3)	3.0 (1.5,5.4)	2.1 (0.9,4.3)	1.3 (0.4,4.2)	0.65
5	0.3 (0.01,1.9)	0.0 (0.0,0.0)	0.0 (0.0,0.0)	0.0 (0.0,0.0)	0.0 (0.0,0.0)	-**	-**
1	0.0 (0.0,0.0)	0.0 (0.0,0.0)	1.0 (0.2,2.9)	0.6 (0.1,2.0)	0.0 (0.0,0.0)	-**	-**
7F	0.0 (0.0,0.0)	0.0 (0.0,0.0)	1.6 (0.5,3.8)	0.0 (0.0,0.0)	0.9 (0.2,2.3)	-**	-**
6A	14.2 (10.2,19.2)	9.5 (6.1,14.0)	8.6 (5.6,12.5)	10.7 (7.6,14.6)	11.1 (7.8,15.3)	0.8 (0.5,1.3)	0.34
19A	2.7 (1.2,5.3)	6.4 (3.8,10.3)	7.6 (4.8,11.4)	6.9 (4.4,10.1)	5.4 (3.2,8.5)	2.2 (0.9,5.1)	0.08
3	2.0 (0.7,4.4)	2.3 (0.8,5.0)	3.0 (1.5,5.6)	1.9 (0.8,4.0)	1.2 (0.3,3.1)	0.6 (0.2,2.2)	0.45
16F	0.3 (0.01,1.9)	6.1 (3.5,9.8)	3.3 (1.6,6.1)	5.8 (3.6,8.8)	3.9 (2.1,6.7)	**12.6 (1.6,97.3)**	**0.015**
10A	0.3 (0.01,1.9)	1.1 (0.2,3.3)	3.6 (1.8,6.5)	1.1 (0.3,2.8)	3.6 (1.9,6.3)	**11.6 (1.5,90.0)**	**0.02**
15A	0.3 (0.01,1.9)	1.1 (0.2,3.3)	3.0 (1.4,5.6)	3.0 (1.5,5.4)	1.8 (0.7,3.9)	5.7 (0.7,47.6)	0.11
15B	1.4 (04,3.5)	1.5 (0.4,3.9)	2.3 (0.9,4.7)	3.0 (1.0,5.4)	1.5 (0.5,3.5)	1.2 (0.3,4.4)	0.82
13	0.7 (0.1,2.4)	3.0 (1.3,6.0)	2.6 (1.1,5.2)	1.4 (0.4,3.2)	3.0 (1.4,5.5)	**4.8 (1.0,22.1)**	**0.05**
11A	3.4 (1.6,6.2)	4.2 (2.1,7.5)	4.0 (2.0,6.9)	5.8 (3.6,8.8)	4.5 (2.5,7.4)	1.4 (0.6,3.2)	0.4
21	2.0 (0.7,4.4)	2.7 (1.1,5.5)	2.6 (1.1,5.2)	2.5 (1.1,4.7)	3.0 (1.4,5.5)	1.6 (0.6,4.4)	0.39
34	2.0 (0.7,4.4)	3.8 (1.8,7.0)	6.3 (3.8,9.8)	1.9 (0.8,4.0)	2.7 (1.2,5.1)	1.4 (0.5,4.0)	0.52
20	1.4 (0.4,3.5)	3.8 (1.8,7.0)	0.7 (0.1,2.4)	1.6 (0.6,3.6)	1.2 (0.3,3.1)	0.9 (0.2,3.8)	0.92
8	0.7 (0.1,2.4)	1.5 (0.4,3.9)	4.0 (2.0,6.9)	0.8 (0.2,2.4)	0.0 (0.0,0.0)		

33F	0.7 (0.1,2.4)	1.1 (0.2,3.3)	0.7 (0.08,2.38)	0.6 (0.1,2.0)	0.6 (0.1,2.2)	0.9 (0.1,6.7)	0.94
22F	0.7 (0.1,2.4)	0.4 (0.01,2.1)	1.0 (0.2,2.9)	0.3 (0.01,1.5)	0.6 (0.1,2.2)	0.9 (0.1,67)	0.94

* P values are for crude differences in the serotype-specific carriage prevalence in the final survey (Year 5 - 2020) compared to the baseline survey (Year 1 - 2016) using Chi square test. P values for bolded ORs are <0.05 and are two-sided. **ORs could not be calculated because carriage was not observed for these serotypes in either Year 1 or Year 5 or both.

Supplement Table 3: Annual serotype-specific carriage prevalence and crude odds ratio (OR) and 95% CI from logistic regression comparing carriage in the final survey (Year 4) to the baseline survey (year 1) of VTs and the commonest NVTs among children aged <5 years in Pakoto, the urban site

Children aged <5 years in Pakoto (urban)						
	Prevalence (95% CI)					
Serotype	Year 1	Year 2	Year 3	Year 4	Crude OR (95% CI)	P value*
19F	13.7 (10.1,18.3)	4.5 (2.3,8.1)	7.0 (4.1,11.2)	3.8 (1.5,7.8)	**0.37 (0.16,0.84)**	**0.018**
6B	10.5 (7.3,14.5)	7.8 (4.7,12.2)	6.6 (3.8,10.7)	3.8 (1.5,7.8)	0.51 (0.22,1.18)	0.12
23F	7.5 (4.8,11.0)	4.1 (2.0,7.5)	2.1 (0.7,4.8)	1.1 (0.1,3.9)	**0.20 (0.05,0.86)**	**0.03**
14	3.6 (1.9,6.3)	0.8 (0.1,3.0)	0.8 (0.1,3.0)	1.6 (0.3,4.7)	0.67 (0.18,2.42)	0.54
9V	1.5 (0.5,3.5)	1.6 (0.5,4.2)	0.4 (0.01,2.29)	0.5 (0.01,3.0)	0.54 (0.06,4.66)	0.57
4	0.9 (0.2,2.62)	1.2 (0.3,3.6)	0.0 (0.0,0.0)	0.5 (0.01,3.0)	0.90 (0.09,8.79)	0.93
18C	0.0 (0.0,0.0)	2.9 (1.2,5.9)	2.5 (0.9,5.4)	0.5 (0.01,3.0)	-**	-**
6A	7.8 (5.1,11.4)	11.5 (7.6,16.6)	7.4 (4.4,11.7)	2.7 (0.9,6.3)	0.49 (0.18,1.33)	0.16
19A	5.4 (3.2,8.5)	8.6 (5.3,13.2)	7.8 (4.7,12.2)	7.6 (4.1,12.7)	**2.30 (1.09,4.82)**	**0.03**
3	2.4 (1.0,4.7)	1.6 (0.5,4.2)	1.7 (0.5,4.2)	1.1 (0.1,3.9)	0.67 (0.14,3.22)	0.62
15B	3.0 (1.4,5.5)	4.1 (2.0,7.5)	4.5 (2.3,8.1)	4.9 (2.2,9.2)	**2.59 (1.02,6.58)**	**0.05**
16F	1.2 (0.3,3.1)	2.5 (0.9,5.4)	4.1 (2.0,7.6)	3.8 (1.5,7.8)	**5.03 (1.44,17.62)**	**0.01**
11A	2.7 (1.2,5.1)	2.5 (0.90,5.35)	5.8 (3.2,9.7)	3.2 (1.2,7.1)	1.86 (0.64,5.37)	0.25
15A	0.0 (0.0,0.0)	0.8 (0.1,3.0)	0.4 (0.01,3.0)	3.2 (1.2,7.1)	-**	-**
23B	1.2 (0.3,3.1)	0.0 (0.0,0.0)	0.4 (0.01,3.0)	2.2 (0.6,5.5)	2.78 (0.68,11.37)	0.15
38	0.9 (0.2,2.6)	0.4 (0.01,3.0)	0.0 (0.0,0.0)	2.2 (0.6,5.5)	3.72 (0.82,16.98)	0.09
10A	0.6 (0.1,2.2)	1.6 (0.5,4.2)	1.2 (0.3,3.6)	1.1 (0.1,3.9)	2.74 (0.38,19.79)	0.32
37	0.3 (0.01,1.7)	0.8 (0.10,3.0)	0.0 (0.0,0.0)	1.1 (0.1,3.1)	5.51 (0.49,61.62)	0.17
35B	2.4 (1.03,4.7)	0.4 (0.01,3.0)	0.4 (0.01,3.0)	0.5 (0.01,3.0)	0.33 (0.04,2.69)	0.30
20	0.6 (0.1,2.2)	0.4 (0.01,3.0)	1.2 (0.3,3.6)	0.5 (0.01,3.0)	1.36 (0.12,15.17)	0.80
12F	0.3 (0.01,1.7)	0.4 (0.01,3.0)	0.8 (0.1,3.0)	0.5 (0.01,3.0)	2.73 (0.17,44.11)	0.48
22F	0.0 (0.0,0.0)	0.0 (0.0,0.0)	0.0 (0.0,0.0)	0.5 (0.01,3.0)	-**	-**

15C	0.3 (0.01,1.7)	3.3 (1.4,6.5)	3.7 (1.7,7.0)	0.5 (0.01,3.0)	2.73 (0.17,44.11)	0.48
13	0.3 (0.01,1.7)	0.8 (0.1,3.0)	0.4 (0.01,3.0)	0.0 (0.0,0.0)	-**	-**
21	1.2 (0.3,3.1)	0.4 (0.01,2.3)	1.7 (0.5,4.2)	0.0 (0.0,0.0)	-**	-*8

* P values are for crude differences in the serotype-specific carriage prevalence in the final survey (Year 5 - 2020) compared to the baseline survey (Year 1 - 2017) using Chi square test. P values for bolded ORs are <0.05 and are two-sided. **ORs could not be calculated because carriage was not observed for these serotypes in either Year 1 or Year 4 or both.

Supplement Table 4: Annual crude serotype-specific carriage prevalence and crude odds ratio (OR) and 95% CI from logistic regression comparing carriage in the final survey (Year 5) to the baseline survey (year 1) of VTs and the commonest NVTs among persons aged ≥5 years in Kumbotso, the rural site

Persons aged ≥5 years in Kumbotso (rural)							
	Prevalence (95% CI)						
Serotype	Year 1	Year 2	Year 3	Year 4	Year 5	Crude OR (95% CI)	P value*
19F	5.0 (3.3,7.2)	3.3 (2.0,5.0)	1.2 (0.5,2.3)	2.3 (1.3,3.8)	1.6 (0.8,3.0)	**0.3 (0.1,0.6)**	**0.001**
23F	3.4 (2.1,5.3)	2.4 (1.4,4.0)	2.9 (1.8,4.4)	2.5 (1.4,4.1)	1.6 (0.8,3.0)	**0.4 (0.2,0.9)**	**0.025**
9V	2.2 (1.2,3.8)	1.5 (0.7,2.8)	0.7 (0.2,1.7)	0.8 (0.3,1.9)	0.2 (0,0.9)	**0.1 (0.01,0.5)**	**0.008**
4	1.6 (0.7,2.9)	2.8 (1.6,4.4)	1.3 (0.6,2.5)	0.3 (0.04,1.2)	1.5 (0.7,2.8)	0.9 (0.3,2.2)	0.74
6B	1.6 (0.7,2.9)	1.6 (0.8,3.0)	1.3 (0.6,2.5)	1.0 (0.4,2.1)	1.3 (0.6,2.5)	0.8 (0.3,2.0)	0.57
18C	1.4 (0.6,2.7)	1.3 (0.6,2.6)	2.6 (1.5,4.1)	1.3 (0.6,2.6)	2.3 (1.2,3.8)	1.5 (0.6,3.6)	0.36
14	1.0 (0.4,2.2)	0.3 (0.04,1.2)	0.4 (0.1,1.3)	0.7 (0.2,1.7)	0.8 (0.3,1.9)	0.7 (0.2,2.3)	0.57
7F	0.5 (0.1,1.5)	0.0 (0.0,0.0)	0.9 (0.3,1.9)	0.8 (0.3,1.9)	0.5 (0.1,1.4)	0.9 (0.2,4.3)	0.85
1	0.3 (0.04,1.2)	0.0 (0.0,0.0)	1.2 (0.5,2.3)	1.2 (0.5,2.4)	0.0 (0.0,0.0)	-**	-**
5	0.0 (0.0,0.0)	0.0 (0.0,0.0)	0.3 (0.3,1.0)	0.0 (0.0,0.0)	0.0 (0.0,0.0)	-**	-**
3	4.3 (2.8,6.3)	5.0 (3.4,7.2)	3.6 (2.3,5.3)	3.1 (1.9,4.9)	6.4 (4.6,8.9)	1.4 (0.8,2.4)	0.20
6A	4.1 (2.6,6.1)	2.3 (1.2,3.8)	2.7 (1.7,4.3)	3.0 (1.8,4.7)	1.8 (0.88,3.2)	0.8 (0.3,2.0)	0.57
19A	0.5 (0.1,1.5)	1.3 (0.6,2.6)	2.2 (1.2,3.6)	1.9 (0.9,3.2)	2.4 (1.4,4.0)	**4.4 (1.3,15.3)**	**0.02**
34	2.9 (1.7,4.7)	3.3 (2.0,5.0)	3.2 (2.0,4.8)	2.6 (1.5,4.3)	3.9 (2.5,5.8)	1.2 (0.6,2.3)	0.54
11A	2.8 (1.6,4.5)	2.8 (1.6,4.4)	2.9 (1.8,4.4)	2.3 (13,3.8)	3.7 (2.4,5.6)	1.2 (0.7,2.4)	0.52
16F	1.2 (0.5,2.5)	2.4 (1.4,4.0)	3.3 (2.1,5.0)	3.1 (1.9,4.9)	3.7 (2.4,5.6)	**2.9 (1.2,6.9)**	**0.015**
10A	1.4 (0.6,2.7)	1.3 (0.6,2.6)	2.6 (1.5,4.1)	2.3 (1.3,3.8)	3.5 (2.2,5.4)	**2.4 (1.1,5.5)**	**0.035**
37	0.5 (0.1,1.5)	1.3 (0.6,2.6)	1.6 (0.8,2.8)	2.6 (1.5,4.3)	2.7 (1.6,4.4)	**5.0 (1.5,17.2)**	**0.011**
8	1.4 (0.6,2.7)	2.8 (1.6,4.4)	4.8 (3.3,6.7)	3.0 (1.8,4.7)	1.8 (0.9,3.2)	1.2 (0.5,3.0)	0.73
21	2.2 (1.2,3.8)	1.3 (0.6,2.6)	2.5 (1.4,3.9)	1.6 (0.8,3.01)	1.5 (0.7,2.8)	0.6 (0.3,1.4)	0.22
13	1.4 (0.6,2.7)	2.8 (1.6,4.4)	1.9 (1.0,3.2)	2.5 (1.4,4.1)	1.3 (0.6,2.5)	0.9 (0.3,2.3)	0.75
35B	2.1 (1.1,3.6)	0.0 (0.0,0.0)	0.1 (0.0,0.8)	0.8 (03,1.9)	0.8 (0.3,1.9)	**0.4 (0.1,1.0)**	**0.05**
15B	1.2 (0.5,2.5)	0.7 (0.2,1.7)	0.7 (0.2,1.7)	0.7 (0.2,1.7)	0.6 (0.2,1.7)	0.5 (0.1,1.7)	0.25

22F	0.9 (0.9,2.0)	1.0 (0.4,2.1)	0.9 (0.3,1.9)	0.5 (0.1,1.4)	0.5 (0.1,1.4)	0.5 (0.1,2.2)	0.36
33F	0.5 (0.1,1.5)	1.6 (0.8,3.0)	1.2 (0.5,2.3)	0.5 (0.1,1.4)	0.2 (0.0,0.9)	0.3 (0.03,2.7)	0.28

* P values are for crude differences in the serotype-specific carriage prevalence in the final survey (Year 5 - 2020) compared to the baseline survey (Year 1 - 2016) using Chi square test. P values for bolded ORs are <0.05 and are two-sided. **ORs could not be calculated because carriage was not observed for these serotypes in either Year 1 or Year 5 or both.

Supplement Table 5: Annual serotype-specific carriage prevalence and crude odds ratio (OR) and 95% CI from logistic regression comparing carriage in the final survey (Year 4) to the baseline survey (year 1) of VTs and the commonest NVTs among persons aged ≥5 years in Pakoto, the

Persons aged ≥5 years in Pakoto (urban)						
	Prevalence (95% CI)					
Serotype	Year 1	Year 2	Year 3	Year 4	Crude OR (95% CI)	P value*
23F	3.2 (1.9,5.0)	0.9 (0.3,1.9)	1.6 (0.80,2.86)	1.2 (0.52,2.37)	0.33 (0.14,0.78)	0.01
19F	2.7 (1.6,4.4)	3.0 (1.9,4.6)	2.6 (1.55,4.13)	0.9 (0.33,1.96)	0.30 (0.11,0.78)	0.01
6B	2.4 (1.3,4.0)	1.9 (1.0,3.2)	2.2 (1.22,3.59)	0.8 (0.24,1.75)	0.29 (0.10,0.81)	0.02
14	1.4 (0.6,2.7)	0.6 (0.2,1.5)	0.6 (0.16,1.49)	0.2 (0,0.84)	0.10 (0.01,0.82)	0.03
18C	1.2 (0.5,2.5)	3.2 (2.0,4.8)	1.5 (0.70,2.67)	1.1 (0.42,2.17)	0.84 (0.29,2.43)	0.74
9V	0.7 (0.2,1.7)	1.1 (0.5,2.3)	0.2 (0,0.81)	0.2 (0,0.84)	0.21 (0.02,1.87)	0.16
4	0.7 (0.2,1.7)	1.1 (0.5,2.3)	0.7 (0.24,1.69)	2.0 (1.04,3.34)	2.84 (0.91,8.84)	0.07
7F	0.3 (0.04,1.2)	0.3 (0.03,1.0)	0.2 (0,0.81)	1.4 (0.62,2.57)	3.90 (0.83,18.27)	0.08
5	0.0 (0.0,0.0)	0.1 (0.0,0.8)	0.0 (0.0,0.0)	0.2 (0,0.84)	-**	-**
1	0.0 (0.0,0.0)	0.1 (0.0,0.8)	0.0 (0.0,0.0)	0.0 (0.0,0.0)	-**	-**
6A	2.7 (1.6,4.4)	2.3 (1.3,3.7)	2.2 (1.2,3.6)	1.2 (0.5,2.4)	3.90 (0.83,18.27)	0.08
3	1.9 (0.9,3.3)	3.7 (2.4,5.5)	1.7 (0.9,3.0)	2.3 (1.3,3.7)	1.16 (0.52,2.58)	0.72
19A	1.5 (0.7,2.9)	1.6 (0.8,2.8)	2.3 (1.3,3.8)	2.1 (1.2,3.5)	1.33 (0.56,3.14)	0.52
11A	2.2 (1.2,3.8)	3.0 (1.9,4.6)	3.2 (2.0,4.8)	2.6 (1.5,4.1)	1.11 (0.53,2.34)	0.79
16F	1.2 (0.5,2.5)	3.2 (2.0,4.8)	2.9 (1.8,4.5)	1.1 (0.4,2.2)	0.84 (0.29,2.43)	0.74
23B	0.9 (0.3,20)	0.3 (0.03,1.0)	0.3 (0.04,1.1)	1.7 (0.8,3.0)	1.89 (0.65,5.55)	0.24
34	0.3 (0.04,1.2)	1.3 (0.6,2.4)	1.6 (0.8,2.9)	1.1 (0.4,2.2)	3.01 (0.62,14.65)	0.17
21	0.2 (0.01,1.0)	1.1 (0.5,2.3)	0.7 (0.2,1.7)	1.1 (0.4,2.2)	6.04 (0.74,49.58)	0.09
15B	0.7 (0.2,1.7)	2.4 (1.4,3.9)	1.0 (0.4,2.1)	0.9 (0.3,2.0)	1.27 (0.35,4.57)	0.71
12F	0.3 (0.04,1.2)	0.0 (0.0,0.0)	0.0 (0.0,0.0)	0.9 (0.3,2.0)	2.57 (0.51,12.88)	0.25
8	0.2 (0.01,1.0)	1.1 (0.5,2.3)	1.2 (0.5,2.3)	0.9 (0.3,2.0)	5.16 (0.62,43.24)	0.13
15C	0.3 (0.04,1.2)	1.1 (0.5,2.3)	0.3 (0.04,1.1)	0.9 (0.3,2.0)	2.57 (0.51,12.86)	0.25
10A	0.3 (0.04,1.2)	0.6 (0.2,1.5)	1.5 (0.7,2.7)	0.6 (0.2,1.5)	1.70 (0.31,9.36)	0.54

22F	0.2 (0.01,1.0)	0.1 (0.01,0.8)	0.3 (0.04,1.1)	0.5 (0.1,1.3)	2.55 (0.26,24.70)	0.42
13	0.7 (0.2,1.7)	2.2 (1.2,3.5)	0.9 (0.3,1.9)	0.3 (0.04,1.1)	0.42 (0.08,2.30)	0.32

* P values are for crude differences in the serotype-specific carriage prevalence in the final survey (Year 5 - 2020) compared to the baseline survey (Year 1 - 2017) using Chi square test. P values for bolded ORs are <0.05 and are two-sided. **ORs could not be calculated because carriage was not observed for these serotypes in either Year 1 or Year 4 or both.

Supplement Table 6: Annual coverage of PCV10 among children aged <5 years.

PCV10 coverage						
Dose	1 Dose	95% (CI)	2 Doses*	95% (CI)	3 Doses	95% (CI)
Kumbotso (rural)						
Time since PCV10 introduction						
Year 1	8.04	6.89, 9.32	7.39	6.29, 8.63	6.56	5.52, 7.73
Year 2	34.3	28.10, 41.00	31.90	25.90, 38.5	27.90	22.30, 34.20
Year 3	47.37	43.5, 51.27	41.62	37.87, 45.46	34.64	31.10, 38.36
Year 4	70.38	66.79, 73.74	62.44	58.72, 66.03	54.20	50.45, 57.90
Year 5	90.33	87.88, 92.33	84.13	81.19, 86.68	76.33	73.02, 79.36
Pakoto (urban)						
Time since PCV10 introduction						
Year 1	15.54	13.48, 17.82	15.00	12.98, 17.25	14.78	12.77, 17.01
Year 2	45.2	35.40, 54.80	48.00	39.30, 65.2	55.40	47.30, 63.20
Year 3	65.95	61.76, 69.91	61.35	57.10, 65.43	57.36	53.16, 61.46
Year 4	96.67	94.99, 97.8	94.40	92.3, 95.96	92.28	89.93, 94.13

Coverage for Year 1 and Year 2 were estimated using a birth cohort analysis of children observed between Year3 and Year 5 in Kumbotso, and Year 3 and Year 4 in Pakoto.
* PCV10 coverage used in the analysis of relationship between VT carriage and PCV10 uptake in Figure 3 of the main text.

Supplement Table 7: Carriage prevalence (95% CI) of serotypes contained in different PCV formulations in the baseline and final surveys among children aged <5 years and persons aged ≥5 years in the rural and urban sites

		Kumbotso (rural)		Pakoto (urban)	
Formulation	Age group	2016	2020	2017	2020
PCV10 GSK[1]	<5 years	42.0 (36.5,47.8)	22.0 (17.8,26.8)	37.6 (32.6,42.9)	12.0 (8.0,17.5)
	≥5 years	17.0 (14.2,20.3)	9.9 (7.8,12.5)	12.6 (10.1,15.5)	7.7 (5.9,9.9)
PCV10 SII[2]	<5 years	54.4 (48.7,60.0)	35.4 (30.5,40.7)	21.1 (15.8,27.6)	53.1 (47.8,58.4)
	≥5 years	18.7 (15.8,22.1)	10.1 (8.0,12.8)	7.9 (6.1,10.3)	18.7 (15.7,22.0)
PCV13[3]	<5 years	60.8 (55.1,66.2)	39.3 (34.2,44.7)	53.1 (47.8,58.4)	23.2 (17.7,29.9)
	≥5 years	25.9 (22.5,29.7)	20.3 (17.3,23.6)	18.7 (15.7,22.0)	13.2 (10.8,16.0)
PCV15[4]	<5 years	62.2 (56.5,67.5)	40.5 (35.4,45.9)	53.1 (47.8,58.4)	23.8 (18.2,30.4)
	≥5 years	27.3 (23.8,31.1)	20.9 (17.9,24.3)	18.8 (15.9,22.2)	13.7 (11.3,16.5)
PCV20[5]	<5 years	67.9 (62.4,73.0)	51.7 (46.3,57.0)	59.7 (54.4,64.8)	34.1 (27.6,41.2)
	≥5 years	34.0 (30.3,38.0)	31.1 (27.6,34.8)	22.6 (19.4,26,1)	19.5 (16.7,22.7)

[1]GSK 10-valent PCV; [2]Serum Institute of India 10-valent PCV; [3]Pfizer 13-valent PCV; [4]Merck 15-valent PCV; [5]Pfizer 20-valent PCV

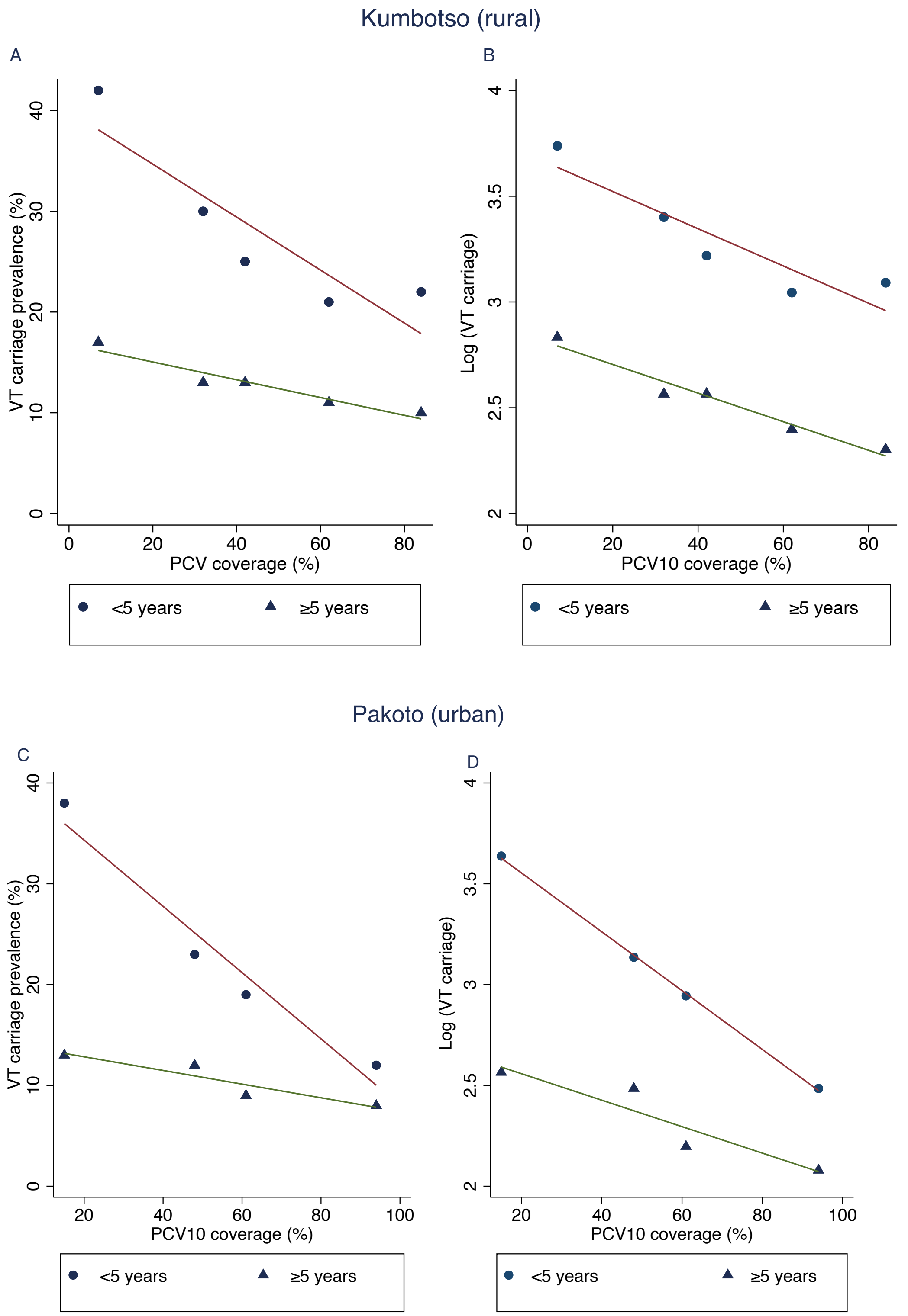

Supplementary Fig. 3: **Relationship between VT carriage and PCV10 coverage.** Graph comparing model fit of linear and non-linear (log-linear) relationship between changes in VT carriage and coverage with 2 doses of PCV10 in Kumbotso (A-B, top) and Pakoto (C-D, bottom).

Using the Akaike Information Criterion (AIC), the non-linear model had a better fit for children aged <5 years compared to the linear model. AIC values for the linear and log-linear models in children aged <5 years are -3.14

and -14.00 in Kumbotso and -3.98 and -17.68 in Kumbotso. The respective AIC values for persons aged ≥5 years are -32.10 and -35.67 in Kumbotso and -23.69 and 12.61 in Pakoto.
In Figure S3 (left), the gradient (95% CI) and R2 for each line of the linear regression are: Kumbotso age <5 years -0.26 (-0.50- -0.03), 0.73; Kumbotso age ≥5 years -0.09 (-0.13- -0.04), 0.93; Pakoto age <5 years -0.33 (-0.54- - 0.11), 0.80; Pakoto age ≥5 years -0.07 (-0.10- -0.04), 0.97.

In Figure S3 (right), the gradient (95% CI) and R2 for each line of the log-linear regression (on the log scale) are: Kumbotso age <5 years -0.0088 (-0.016- -0.0017), 0.83; Kumbotso age ≥5 years -0.0068 (-0.0093- -0.0042), 0.94; Pakoto age <5 years -0.0146 (-0.0157- - 0.013), 0.99; Pakoto age ≥5 years -0.066(-0.014- -0.013), 0.86.

Supplement Table 8: Serotypes included in the different PCV formulations

	Serotypes included																			
Formulation	1	3	4	5	6A	6B	7F	8	9V	10A	11A	12F	14	15B	18C	19A	19F	22F	23F	33F
PCV10[1]	●	●		●		●	●		●				●		●		●		●	
SII-PCV[2]	●	●			●	●	●		●				●			●	●		●	
PCV13[3]	●	●	●	●	●	●	●		●				●		●	●	●		●	
PCV15[4]	●	●	●	●	●	●	●		●				●		●	●	●	●	●	●
PCV20[5]	●	●	●	●	●	●	●	●	●	●	●	●	●	●	●	●	●	●	●	●

[1]GSK 10-valent PCV; [2]Serum Institute of India 10-valent PCV; [3]Pfizer 13-valent PCV; [4]Merck 15-valent PCV; [5]Pfizer 20-valent PCV
Dotted cells represent serotypes included in each vaccine

Supplement Table 9: Annual diversity of serotypes by site, age and year of survey

Age	Year of survey	Kumbotso (rural)		Pakoto (urban)	
		Simpson's diversity index	No. of serotypes identified	Simpson's diversity index	No. of serotypes identified
< 5 years					
	2016	0.976	41		
	2017	0.974	39	0.875	32
	2018	0.978	46	0.831	30
	2019	0.979	49	0.839	29
	2020	0.979	49	0.751	27
≥5 years					
	2016	0.982	55		
	2017	0.981	54	0.817	45
	2018	0.984	62	0.889	41
	2019	0.985	65	0.842	46
	2020	0.984	61	0.883	46

Strain (serotype) diversity was greater in Kumbotso (rural) than Pakoto (urban) and was marginally greater in older persons, but the diversity index did not vary substantially over time after introduction of the vaccine.
