# Supplementary material for: The impact of introduction of the 10-valent pneumococcal conjugate vaccine on pneumococcal carriage in Nigeria
Source: Nat Commun. 2023 May 9;14:2666. doi: 10.1038/s41467-023-38277-z (PMC10169786; doi:10.1038/s41467-023-38277-z)
Supplement: Supplementary file 3 — Description of Additional Supplementary Files [file 41467_2023_38277_MOESM3_ESM.pdf]

Description of Additional Supplementary Files

File Name: Supplementary Data 1
Description: Serotype-specific carriage data. File includes numbers, prevalence (and 95% CI) of all serotypes identified across all surveys in the two sites.

File Name: Supplementary Data 2
Description: Annual PCV10 coverage. File contains anonymised data on receipt of one, two and 3 doses of PCV10, year of survey and year of birth of participants in the two sites.

File Name: Supplementary Data 3
Description: PCV10 coverage and VT carriage prevalence. File contains aggregate data on coverage with two doses of PCV10 (and 95% CI) and VT carriage prevalence (and 95% CI) used for analysis of the linear and exponential relationship between PCV coverage and VT carriage.
